# Pain communication in children with autism spectrum disorder: A scoping review

**DOI:** 10.1002/pne2.12115

**Published:** 2023-10-13

**Authors:** Ensa Johnson, Karen van Zijl, Ariné Kuyler

**Affiliations:** ^1^ Department of Inclusive Education, College of Education University of South Africa Pretoria South Africa; ^2^ School of the Arts: Visual Arts University of South Africa Pretoria South Africa

**Keywords:** autism spectrum disorder (ASD), home, hospital, pain communication, pain expression, pediatric pain, response, school

## Abstract

Children with autism spectrum disorder (ASD) experience social interaction and communication challenges and often display repetitive, restricted patterns of behavior, activities, and interests. The concept of pain is regarded as one of the most complex human stressors due to its subjective and personal nature and the influences of multiple internal and external factors. Due to the complexity of this disorder, it remains concerning how children with ASD communicate their pain and how observers (i.e., parents, carers, and health care practitioners) respond to these children's pain communication. This scoping review aimed to identify how children with ASD communicate or express their pain. Ten studies met the inclusion criteria for further data extraction. Through reflexive thematic analysis, two main themes were identified: verbal and nonverbal responses used by children with ASD to communicate their pain that could influence pain assessment and management strategies. This review highlighted that children with ASD utilized various verbal and nonverbal methods to communicate their pain experiences and that these methods differed compared to children without disabilities. Furthermore, this review emphasizes the importance of holistic pain assessment strategies as well as additional pictorial support for children with ASD. This review recommends that future research should focus on understanding how the inclusion of different stakeholders in pain assessment for children with ASD, can contribute to holistic pain assessment.

## 1 INTRODUCTION

Autism spectrum disorder (ASD) is defined as a heterogeneous neurodevelopmental disorder including challenges with social interaction, communication in various contexts, and the presence of repetitive, restricted patterns of behavior, activities, and interests.^1^, ^2^ For a diagnosis, all of these challenges are typically present but can be experienced in varying levels of severity.^3^ A wide range of behavioral challenges (e.g., self‐harm, anxiety), as well as medical conditions (e.g., gastrointestinal tract problems, epilepsy), are associated with a diagnosis of ASD and may result in frequent pain experiences.^2^, ^4^ These pain experiences—either chronic or acute—may cause distress and increased anxiety for children with ASD.^5^, ^6^, ^7^

Pain‐related anxiety and pain experiences in children with ASD may be exacerbated due to their inability to express or communicate their pain verbally.^6^ Costello et al.^8^ proposed the term communicative vulnerability when referring to patients who experience challenges and reduced capability in expressive or receptive communication. These communication challenges could be either temporary such as reduced verbal communication due to medical interventions or permanent, which is due to severe communication disabilities coexisting with disabilities including ASD and cerebral palsy.^9^ Subsequently, observers (i.e., parents, carers, and health care practitioners) may be unaware or unprepared to support and adapt practices to accommodate children with ASD who may need alternative communication support.^7^

Communicative vulnerability may greatly impact pain management in the pediatric population.^5^ This may be as a result of children experiencing difficulties in conceptualizing pain, but also due to the subjective nature and the individual experiences of pain.^10^ Pain is a complicated multifaceted integration of three dimensions, namely sensory, affective, and cognitive.^11^ These dimensions interact to regulate pain experiences and activate a wide network of brain regions.^11^ Pain regulation by children is considered a complex human stressor due to its subjective and individualized nature, influenced by multiple internal and external factors.^12^ Furthermore, the concepts of pain and suffering go well beyond that of a simple sensory experience due to the intense impact of the emotional component of pain.^12^ As previously mentioned, anxiety is a behavioral challenge for children with ASD, closely associated with pain perception and threshold.^13^ It is difficult for children to avoid feelings of anxiety and distress, even in medical procedures that might seem minor to an adult. To the child, a seemingly benign medical examination may be experienced as distressing. This emotional context can influence the individual's perceived pain intensity.^13^ To identify and assess the individual's perceived pain intensity, self‐reports are often regarded as the gold standard.^14^, ^15^ However, for a child with ASD presenting with a communicative vulnerability, alternative pain assessment tools may be required as opposed to those necessitating verbal input from the child.^5^ Moore^16^ proposed that during pain assessment in ASD, differences in the child's self‐report of pain (i.e., using a colorful faces pain scale to communicate the pain experience) and observational proxy reports of these children's experiences by observers or caregivers may occur. These reporting differences of the pain experiences of children with ASD emphasize the importance of using observational pain assessment tools that focus on behavioral responses in addition to other pain scales.

Children with ASD are vulnerable to mismanagement of their pain by observers due to their reduced verbal self‐reporting.^17^, ^18^, ^19^ As observers, such as health care practitioners, spend limited time with these children, a common misconception is that children with ASD may have either reduced pain sensation thresholds (hyposensitivity) or high pain sensitivity thresholds (hypersensitivity).^20^ Children with ASD tend to express their pain differently than their peers without disabilities, and observers may not be familiar with these behaviors. Though, according to Nader et al.,^20^ in their study of 43 children (21 with ASD, 22 without disabilities), both groups presented with similar behavioral responses to pain during needle procedures, apart from more pronounced facial expressions in children with ASD. Tordjman et al.^21^ further reported the presence of physiological changes, such as elevated heart rate and higher levels of Plasma‐B endorphin, even though behavioral reactions of children with ASD were absent during pain experiences caused by needle procedures. Observers should therefore be aware that children with ASD may communicate their pain using verbal as well as physiological and behavioral responses.

This awareness is necessary as communication—a dynamic reciprocal process that involves persons (acting as a sender or receiver)—includes verbal (speech) as well as nonverbal modes (gestures, a shared glance, facial expression).^22^ In any context, effective communication (when the sender is understood by the receiver) is important for appropriate patient care and pain management.^23^ How persons communicate their pain can explain their experiences of pain, although Knoll et al.^24^ emphasize that these concepts are not the same. Pain communication comprises an observable behavioral response of a person to a harmful stimulus, whereas a person's pain experience is intrinsic and includes the intensity of the pain or distress,^25^ as well as the anxiety related to the subjective perception of the harmful stimulus.^12^

Apart from one published literature review focusing on children with ASD and their pain expression in French^26^ (and not available in English), this topic has received limited attention in research over the past 10 years. Due to the complexity of this disorder, it remains a concern of how children with ASD communicate their pain and how observers respond to these children's pain communication in all settings. This scoping review aimed to identify how children with ASD communicate or express their pain.

## 2 METHOD

### 2.1 Search Protocol

A scoping review was conducted to determine the evidence base on how children with ASD communicate their pain. Scoping reviews are typically used to identify and target literature on a specific topic using inclusion and exclusion criteria. It is further used to identify the nature and extent of existing knowledge.^27^ The PRISMA extension for Scoping Reviews (PRISMA‐ScR), the most recent and advanced approach designed to provide guidance for reporting scoping reviews,^28^ was followed to develop the protocol for this review. The PRISMA‐ScR is largely based on the well‐known PRISMA statement and checklist, the Johanna Briggs Institute (JBI) methodological guidance, and other approaches for undertaking scoping reviews.^29^ The review question for this study was identified through the PCC framework: population—children with ASD; concept—pain; outcomes—communication^30^: *How do children with ASD communicate or express their pain?*

### 2.2 Inclusion criteria

Criteria for the inclusion of studies in this review were (i) peer‐reviewed journal articles published from January 2013 to March 2022 (this date was based on searches of the last two reviews by Allely^31^ and Moore^16^ that were done from February 2013 to August 2013 that deem identifying studies before 2013 unnecessary), (ii) written in English (as translations are costly), (iii) involved children (aged 3–17; 11 [years; months]) who were diagnosed with ASD and experienced and expressed pain in various settings (e.g., hospital, home, and school). Proxy reports by caregivers (including parents and carers) were also included. (iv) Quantitative, qualitative, mixed‐method studies, case studies, as well as review studies and commentaries were included to consider any possible design reporting on children with ASD expressing their pain. Studies were excluded if they did not meet the selection criteria (e.g., did not include information on pain expression or communication; wrong population—i.e., adults with ASD, non‐English articles, or gray literature—i.e., book reviews, book chapters, dissertations, theses, web pages).

### 2.3 Search strategy

To identify relevant studies, a pilot search was conducted by the first author between November 2021 and December 2021 in the online library of a higher education institution to confirm search terms for the review. From January 2022 to March 2022, the final searches were made in the following data bases: Academic Search Premium, Academic Search Ultimate, CINAHL, EbscoHost Medline, Health Source Nursing, Lippencot Nursing Journals, and PubMed. A hand search was done via Google Scholar and ResearchGate to ensure that all potential studies were identified. Where the full texts of the selected studies were not available via the institution's online library, requests were made to authors via ResearchGate for the full‐text version. An experienced librarian from the institution supported the researcher in identifying relevant keywords related to the PCC question which was employed in the keyword search. BOOLEAN operators (AND and OR) as well as truncation were used in the search strings.^28^ The keywords string used were (Autism Spectrum Disorder OR Autistic Disorder OR autism or ASD or autistic) AND child* AND pain AND (express* OR communicate*).

### 2.4 Search procedure

**FIGURE 1:**
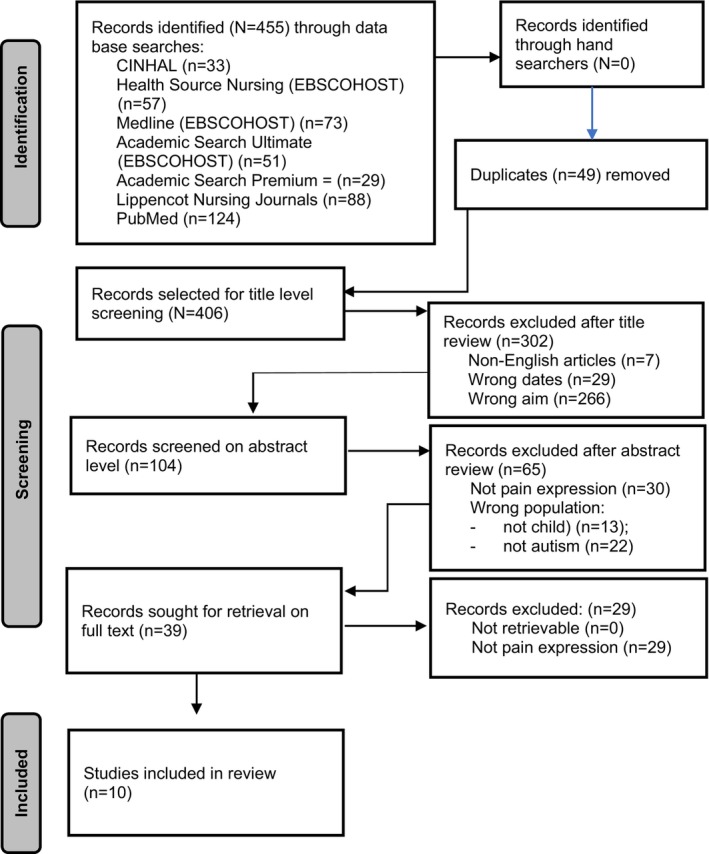
PRISMA‐ScR identification of studies.

Initially, a potential 455 studies published since January 2013 were identified from the seven database searches (see Figure 1). No new studies were identified through hand searches. A total number of 49 duplicates were removed from the potential 455 studies. A total of 406 studies were screened by the first author on title level to determine if they might meet the inclusion criteria as stated earlier whereafter 302 titles were removed (of which seven were not in English). Thereafter, two reviewers (the first author and the third author) independently screened the remaining 104 studies on abstract level to identify potential studies relevant for the current review. The first and third author reviewed the abstracts for all the studies separately, to obtain a reviewer agreement. An 84% rater agreement was obtained initially during abstract level screening, followed by 100% consensus after discussions between all three authors. Following the discussion, minor amendments were made to the screening and data extraction form to remove any further uncertainties during the selection process for the screening of full texts. A total number of 65 studies were excluded on abstract level resulting in an inclusion of 39 studies for full‐text screening. The same process followed for the abstract‐level screening, was followed for the full‐text‐level screening of studies. For full‐text screening an initial rater agreement of 94% was obtained followed by a 100% consensus after discussion. After reviewing the full‐text studies and applying the inclusion and exclusion criteria, the data of only 10 identified and relevant studies were extracted. Since the inclusion criteria stipulated published studies from 2013, no hand searches were done from the two review studies, the data were used as a presented in these two review studies. The authors, however, acknowledge that some data might have been misinterpreted from the original studies. Figure 1 shows the PRISMA‐ScR diagram including the searches from title to full‐text level.

#### 2.4.1 Data extraction

The two reviewers (first and second authors) developed a data extraction form to determine which variables to extract. They extracted the data independently, discussed the results and updated the data extraction form in a continued process. Data were extracted based on study characteristics, for example, authors, year and country, target population, age of ASD participants, number of participants, setting where the research was conducted, aims of the study, design, data collection procedure, self‐report, proxy‐report, observer, materials related to pain expression (assessment) and outcomes relevant to the pain communication or expression of children with ASD.

#### 2.4.2 Data analysis

The 10 full‐text selected studies were uploaded onto ATLAS.ti, a computer‐aided qualitative data analysis software.^32^ Findings from the two review studies were included in the current review without any further analysis of the data. Inductive reflexive thematic analysis was used to analyze the data.^33^ In reflexive thematic analysis, the coding process is an inherent part of the theme development, in that themes are developed through coding.^33^ During the reflexive thematic analysis, the proposed six‐step approach by Braun and Clarke^33^ was followed, namely: (i) familiarization of data and writing familiarization notes; (ii) systematic coding of the data; (iii) generating initial themes derived from coded and collated data; (iv) developing and reviewing themes; (v) refining, defining and naming themes; and (vi) compiling the report.

## 3 RESULTS

A total of 10 studies matched the selection criteria for full text data selection. Table 1 presents the study characteristics, namely, author, year, country, target population; number of participants, age of children, setting where study was conducted, aims, design, data collection procedure, self‐report, proxy report or observer, materials related to pain expression and outcomes of the studies included in this review. These findings reported on study level data present only the information relevant to answer the aims of this review, namely pain communication or expression of children with ASD.

**TABLE 1:** Study‐level data: Characteristics of included studies.

Author; Year; Country	Target population; setting	Age range of children; Total participants	Aim	Design	Data collection procedure	Self‐report; proxy report; observer	Materials related to pain expression	Outcomes
Allely; 2013; n.a	Children with ASD; various settings including hospital, dentist, home, school	1:06 to 21:00 year‐olds (*n* = 1321)	to determine if individuals with ASD are insensitive to examine whether individuals with ASD react or express pain differently	Review	Review study following the PRISMA guidelines.	Self‐report; Proxy report Observer	*Observation scales*: Brief Behavioral Distress Scale (BBDS) Child Facial Action Coding System Revised Manual (CFCS) Observational Scale of Behavioral Distress (OSBD) Pre‐Linguistic Behavioral Pain Reactivity Scale (PL‐BPRS) Non‐Communicating Children's Pain Checklist Revised (NCCPC) *Self‐report scales*: Faces Pain Scale (FPS)/ Faces Pain Scale Revised (FPS‐R) McGill Pain Questionnaire (MPQ) Numeric Rating Scale (NRS) Visual Analogue Scale (VAS)	Significant behavioral responses to pain, for example, facial responses and aggressive behavior were displayed Physiological responses (i.e., heartbeat, sweating) can indicate pain. Apart from 1 of the 10 experimental studies, all reported hypersensitivity. Despite physiological changes (higher heart rate and Plasma‐b endorphin concentrations), some studies reported limited behavioral pain responses. Difficult to shift attention from pain to something that could increase pain and distress. Potential treatment for example., squeezing a ball when in pain can change pain severity. Observers rely on children with ASD's facial responses Use verbal and pictorial scales for the child to respond to a question. Rather than ask how much pain they are feeling, use questions like: How are you? How uncomfortable are you? Previous negative pain experiences influence pain responses and might influence children's responses in various settings (i.e., hospital or clinic, home, school).
2 Clarke; 2015; Ireland	Children with ASD in various settings including hospital and home	15;00–16:00 (*n* = 2)	to present two case studies on how individuals with ASD may experience pain and express pain	Case study	Case presentations	Self‐report; Proxy report	Not mentioned	Persons with ASD may experience sensations in all modalities with raised or lowered thresholds and sometimes in qualitatively abnormal ways. Amplified pain may be a presenting feature of autism, and its unusual character may delay diagnosis when individuals present to general physicians
3 Dubois et al.; 2017; France	Children with ASD in various settings including hospital and home	3;00–18:00 (mean 4:05) (*N* = 35)	to describe the responses to the GED‐DI (French version of the NCCPC) as completed by parents of children with ASD, to study the psychometric qualities of the scale in this population, with an analysis of its internal structure validity.	Quantitative descriptive design	Retrospective data collection where parents recall their children's pain incidences in the past 6 months at home	Proxy report Observer	*Observation scales*: Non‐Communicating Children Pain Checklist [NCCPC] French version ‐ Grille d'Evaluation Douleur‐Déficience Intellectuelle (Cronbach Alpha coefficient >0.70)	Children with ASD expressed their pain similar to children without disabilities through diverse behavioral and expressive responses markers (vocal, facial, activity, and social) that is, crying, gesturing and protecting painful area, seeking comfort and physical closeness. Some behavioral responses are more distinctive and cannot be easily recognizable as behaviors linked to pain, that is, specific sound, irritableness, change in eyes. Physiological signs (i.e., shivering, perspiring) and those related to inactivity (not moving) were less prominent in terms of frequency and intensity.
4 Ely et al.; 2016; United States of America	Verbal children with ASD who were scheduled for surgery or admitted to emergency unit; Parents of the same children; hospital setting	6:00–17:00 (N = 40)	to illuminate barriers to pain assessment in children with an ASD, to describe novel methods to communicate about their pain experience to identify vocabularies that hold meaning with respect to pain to better understand pain from their context.	Qualitative, descriptive design	Semi‐structured interviews with parents	Self‐report; Proxy report	Semi‐structure interview guide with specific pain‐related questions (parents and children) Self‐report: Wong Baker FACES Pain scale (WPFPS) Visual Analogue Scale (VAS) Poker Chip Tool iPad with Doodle or body figure app	Do not continuously ask children about their pain Word choice matters (describe pain rather than use numbers) For self‐report: Wong Baker FACES Pain Scale; VAS; iPad with Doodle or body figure app; Poker Chip Tool Combining analgesics and distraction to alleviate pain Most frequent words: hurt, pain, cry
5 English et al.; 2019; United States of America	Parents of children with ASD and children without disabilities below 10:00 years; hospital setting	<10:00 (*n* = 22)	to assess whether the cries of infants later diagnosed with ASD diagnoses sound different to parents of young children without disabilities; to examine whether parents of children with ASD differ from parents of children without disabilities in how they perceive infant cries.	Quantitative	Existing libraries of cry recordings from a longitudinal study provided cry samples of infants. Cry samples of 1‐month‐old infants were confirmed to be later diagnosed with ASD or children without disabilities.	Proxy report Observer	*Observation scales*: Broad Autism Phenotype Questionnaire (BAPQ); Cry Rating Scale	Parents rated cries of 1‐month‐old infants with later ASD diagnosis as reacting to greater levels of distress, less typical, and higher levels of pain when compared to matched controls. Infants with ASD may be characterized by subtle differences in neurobehavioral responses in very early infancy.
6 Fitzpatrick et al.; 2022; United Kingdom	Children with ASD and ID in a school setting	11;02; 11:04; 12:08 (*n* = 3)	to use a behavioral‐based educational intervention to teach children with ASD and ID to communicate about their pain to identify if pain was present for each participant to facilitate skills teaching when pain was present to determine if there was any reduction in challenging behavior in response to the ability to communicate pain.	Quantitative, experimental design	Initial baseline Probes used to determine if an increase in the communication of pain using principles of applied behavior analysis can be achieved. Components of intervention introduced individually and sequentially: label body parts on iPad, label body parts on self, label and score pain severity using the, and request pain relief; In‐situ training.	Self‐report; Proxy report	*Observation scales*: The Non‐Communicating Children's Pain Checklist‐Postoperative Version (NCCPC‐PV) *Self‐report scales*: Wong Baker FACES Pain Scale (WPFPS)	All participants learned how to report pain by indicating the severity of pain using the Wong Baker FACES Pain Scale or verbalizing the location of pain, and then requesting pain relief. All participants could identify body parts but not on their own bodies. Challenging behavior was displayed when pain scores of 11 or above was present. After intervention lower challenging behavior occurred as children could communicate the severity of pain using the Wong Baker FACES scale
7 Moore; 2015; n.a.	Parents of children with ASD in various settings including hospital and home	2:05–10:07 (*n* = 77)	To examine sensitivity to the pain of individuals with an autism spectrum disorder. to report which considers differences in the subjective experience of pain	Review	Retrospective self‐report proxy report clinical observations to reflect the views of trained professionals of individual cases or small groups in their care	Self‐report Proxy report Observer	*Self‐report/Proxy report scales*: Faces Pain Rating Scale.	Different pain responses observed based on the severity of ASD: hyposensitivity; hypersensitivity; typical responses (but not unresponsive) compared to children without disabilities. Facial responses to pain confirmed during venipuncture Anxiety or distress behaviors due to pain Hyposensitivity Increased self‐injurious or repetitive behavior due to pain Increased heart rate and Plasma‐b endorphin levels
8 Palese et al.; 2021; Italy	Caregivers (parents, teachers, babysitters) of children with ASD school (day time center) and home settings	6:00–17:00 years (mean: 12:08); (*n* = 141)	to assess the validity of the NCCPC‐R in identifying pain in children and adolescents affected by ASD	Quantitative	Phase 1: translation and cultural adaptation of NCCPC‐R; Phase 2 Interviews with caregivers and completion of NCCPC‐R on children's pain episodes by recalling a painful episode of their child	Proxy report Observer	*Observation scales*: Non‐Communicating Children Pain Checklist (NCCPC)	Different caregivers (e.g., professional educators, family members) scored the items of the tool according to the different experiences with episodes of pain of their cared children. In accordance with Craig's Social Communication Model of Pain, the pain was decoded by family members, professional educators or other caregivers (e.g., baby sitters); hence, biases influencing their disposition to attend, recognize and understand the behavioral expressions of pain of their child may have affected the findings.
9 Prosperi et al.; 2019; Italy	Children with ASD (*n* = 85) with GI diagnosis in home setting	2.18–6.11 (mean age 4:10) (*n* = 85)	To investigate the correlation between GI symptoms and the presence and the type of AB reported in the Consensus report in an Italian sample of preschoolers with ASD; To evaluate possible differences in the expression of AB between verbal and nonverbal ASD subjects with GI problems.	Quantitative	Determine the scores of GI symptoms between children with ASD and GI and non‐GI group	Proxy report Observer	*Observational scales*: Associated Behaviors Questionnaire (ABQ) Repetitive Behavior Scale‐Revised (RBS‐R) Griffiths Mental Development Scales‐Extended Revised (GMDS‐ER) Vineland Adaptive Behavior Scales‐Second edition (VABS‐II) Child Behavior Checklist 1.5–5 (CBCL 1.5–5)	“frequent clearing of throat, swallowing and/or tics” (*p* = 0.043), “screaming” (*p* = 0.048), “sighing and/or whining” (*p* = 0.039), “moaning and/or groaning” (*p* = 0.003), and “direct verbalization about pain or stomach” (*p* = 0.015) scores was detected
10 Rattaz et al.; 2013; France	Children with ASD (*n* = 35); Children with ID (*n* = 32); Children without disabilities (*n* = 36) in hospital setting	3:00–8:00 (*n* = 35)	To describe the facial, behavioral, and physiological reactions of children with ASD during venipuncture To compare them to the reactions of children with an intellectual disability and nonimpaired conrol children	Quantitative descriptive design	On the day of the venipuncture, the children were video recorded, and their heart rate was recorded.	Proxy report Observer	*Observation scales*: Child Facial Coding System Non‐Communicating Children Pain Checklist (NCCPC) Heart rate monitor	Children with ASD and the comparison group expressed significant facial, behavioral and physiological reactions during venipuncture. Children with ASD displayed a decrease in facial reactions between the end of the venipuncture and the recovery period. Children with ASD showed stronger behavioral reactions than children without disabilities in the comparison group during the whole procedure. Children with ASD tended to recover more slowly than other children, and although facial expressions of pain decrease right after the end of the procedure, behavioral reactions remain high even after the end of the venipuncture.

### 3.1 Study‐level data

Two review 2013 articles pertinent to the pain experiences of individuals with ASD^16^, ^31^ were included in the current review (see Table 1). The focus of the review by Allely^31^ was on psychological and behavioral pain responses in individuals with ASD. The review by Moore^16^ evaluated evidence that individuals with ASD have different sensitivities to pain stimuli and how individuals with ASD communicate pain to those around them. The current review thus serves as an update of the findings from the reviews by Allely^31^ and Moore.^16^ The other eight studies were a case study (*n* = 1); quantitative (*n* = 6) and qualitative (*n* = 1) studies. Two studies each were conducted in Italy, France, and the United States and one in Ireland and the United Kingdom, respectively. The two included review studies did not report on any country where their included studies took place. Two studies each were published in 2013,^4^, ^31^ 2015,^16^, ^34^ and 2019,^35^, ^36^ whereas one study each was published in 2016,^18^ 2017,^37^ 2021,^38^ and 2022.^39^ Proxy reports (by either parents or caregivers, teachers, and health care professionals) were used in five of the included ten studies^4^, ^35^, ^36^, ^37^, ^38^ while the other five studies included both self‐ and proxy reports and observers.^16^, ^18^, ^31^, ^34^, ^39^ Observation scales and self‐report scales to identify children's pain were also extracted from the included studies. The most frequently used pain assessment materials for self‐report include the Wong Baker FACES Pain Scale (WPFPS),^18^, ^39^ Visual Analogue Scale (VAS)^18^, ^31^ while the Non‐Communicating Children's Pain Checklist (NCCPC)^4^, ^37^, ^39^ were mostly reported as an observation scale for proxy reports. Refer to Table 1 for more information of other observation scales that were used.

### 3.2 Participant‐level data

Table 2 provides an overview of the two identified themes, the subthemes and some examples of codes as obtained from the data related to participant level data. The data revealed themes or patterns of shared meaning that were united by a central idea.^40^ Two main themes were identified, namely verbal (*n* = 7) and nonverbal (*n* = 18) responses by children with ASD when they experience pain (see Table 2). Verbal responses include nonspeech‐like or meaningless sounds, such as vegetative sounds (e.g., moaning, chewing sounds, throat clearing, crying, and screaming). Speech‐like sounds refer to meaning laden verbal responses, such as interjections, verbalizations, and spoken words referred to pain. The spoken words consisted of verbs (pain, hurt), adjectives (describing the pain), and nouns (referring to the pain location on the body). Nonverbal responses encompass physiological responses (such as headaches, sleep disturbances, increased heart rate, and abdominal pain); sensory processing of pain impulses (such as hyposensitivity and hypersensitivity), and communication such as facial expressions, gestures, pointing, and use of a device for distraction during or following a pain experience. Socio‐emotional responses include, for example, aggressive behaviors, increased emotional responses, and repetitive behavior, protection of painful areas, self‐harm, and withdrawal.

**TABLE 2:** Participant‐level data: Themes, subthemes, and examples of codes.

Themes	Subthemes	Codes
*Verbal responses* (*n* = 7) (Refer to vocalizations in response to noxious stimuli and categorized according to nonspeech‐like and speech‐like verbal responses)	Nonspeech‐like (meaningless vocal responses)	
	Vegetative sounds	“sighing, wining, moaning and/or groaning” (Prosperi et et al., 2019)
	Crying	“crying” (Dubois et al., 2017) “cries of infant” (English et al., 2019); “tears” (Palese et al., 2021)
	Throat clearing	“frequent clearing of throat” (Dubois et al., 2017)
	Screaming	“screaming” (Prosperi et al., 2019)
	Speech‐like (meaning‐laden vocal responses)	
	Verbalizations	“Direct verbalization about pain” (Prosperi et al., 2019)
	Words	“hurt”, “pain”; “preferred using words to describe pain rather than numbers” (Ely et al., 2016); “verbalizing the location of pain, requesting pain relief” (Fitzpatrick et al., 2022)
	Interjections	“Ow!” (Allely et al., 2013)
*Nonverbal responses* (*n* = 18) (Refer to behavioral, social, emotional and communicative responses to noxious stimuli)	Physiological responses (physiological reactions of the body to noxious stimuli)	
	Sleep disturbances	“sleep disturbances” (Prosperi et al., 2019); “…would wake up frequently during the night” (Allely et al., 2013)
	Increased heart rate	“The mean heart rate increased significantly from baseline” (Rattaz et al., 2013)
	Headaches	“he complained of excessive noise, did not wish to be touched, and covered his head.” (Allely, et al., 2013)
	Abdominal pain	“… was reported as having long‐ term headaches and abdominal pain.” (Moore et al., 2014)
	Sensory dysregulation (Difficulty regulating sensory input)	
	Hypersensitivity (low pain threshold)	“My child is very sensitive to pain of bumps or cuts or other common hurts.” (Allely et al., 2013); “…these individuals are not insensitive to pain, at least during medical procedures, even if they may react in a different manner than their non‐ASD peers.” (Moore et al., 2014).
	Hyposensitivity (high pain threshold)	Atypical ways in which autistic individuals experience pain, for example, denying pain but describing such noxious stimuli as dental extraction as “discomfort.” (Clarke 2015); “lower intensity of pain” (Allely et al., 2013)
	Atypical sensory processing	“…had sensory abnormalities, for example, ‘he once grabbed a hot frying pan and did not seem to respond in a way typical of someone in pain’”; “lacked sensation and did not feel pain, even when struck hard” (Moore et al., 2014)
	Communication (Intentional communicative responses to noxious stimuli)	
	Facial expression	“Facial activity of a group of ASD children” (Moore et al., 2014); “facial grimacing” (Prosperi et al., 2019)
	Gestures; pointing	“show where the pain was located” (Ely et al., 2016); “pointed to the WBFCS” (Fitzpatrick et al., 2022)
	Use device as distraction	“For many, the iPad provided an alternative method of communicating about pain” (Ely et al., 2016)
	Socio‐emotional responses (social or emotional behavioral responses to noxious stimuli)	
	Increased emotional responses	“greater stress/anxiety/distress after the procedure” (Rattaz et al., 2013)
	Expressed behavioral intent	“abnormal behavioral responses to painful stimuli were highly prevalent” (Allely et al., 2013); “behavioral responses seemed to be higher in children with ASD after the venepuncture” (Rattaz et al., 2013); “challenging behavior” (Fitzpatrick et al., 2022)
	Increased repetitive behavior	“rocking unusually” (Allely et al., 2013)
	Inactivity	“being less active” (Allely et al., 2013); “lack of reactivity” (Dubois et al., 2017)
	Self‐harm	“She became sad and began to superficially cut her forearms” (Clarke 2015); “The sudden appearance of self‐ and other‐directed aggression…” (Prosperi et al., 2019)
	Aggressive behavior	“aggressive behaviors” (Allely et al., 2013) “out‐directed aggression” (Prosperi et al., 2019)
	Protection of painful area	“Protecting painful area or applying pressure to abdomen” (Prosperi et al., 2019)
	Withdrawal	“social withdrawal” (Clarke 2015)

## 4 DISCUSSION

This review aimed to identify how children with ASD communicate or express their pain experiences. From the findings of this study only a small number of published studies are available regarding pain communication among children with ASD. From the many possible studies published since 2013 (*N* = 455), only 10 studies matched the inclusion criteria of this review. Children with ASD may have frequent pain experiences due to potential risk of injury during self‐harm behavior (e.g., head banging and scratching), aggressive behaviors, and medical conditions (e.g., gastrointestinal tract problems, epilepsy) associated with the ASD population.^3^ It is therefore necessary to identify their unique modes of pain communication.

The studies included in this review are all from countries in the Global North, such as Italy,^35^, ^38^ France,^4^, ^37^ Ireland,^34^ United Kingdom,^39^ and the United States^18^, ^36^ with no from the Global South. The Global South refers to countries that were previously referred to as underdeveloped and have faced colonialization by the Global North and also referred to as “low‐income” economies.^41^ The latter include, according to the World Bank classification,^42^ low‐income countries (such as Mozambique, Sudan), low‐to‐middle income countries (such as Zimbabwe, India, and Eswatini), and upper‐to‐middle income countries (such as South Africa, Brazil, and Argentina). Despite the drive to combat health disparity as included the sustainable developmental goals, persons with disabilities, including those with ASD, living in these lower income countries still experience challenges when accessing health care.^43^, ^44^ Cultural and attitudinal barriers,^45^ logistical barriers (lack of transport; rural areas),^44^ financial barriers (poverty, limited resources),^45^ environmental barriers (lack of ramps to buildings) together with the lack of policy implementation^46^ in these countries are among others contributing factors that children with ASD and their parents may have to deal with when accessing health care. Nonetheless, even though no studies from the Global South were included in this review, the findings of this study will enable health care professionals working in the Global South to include both verbal and nonverbal expressions from children with ASD as pain assessment strategies.

Interesting to note is that not one of the included studies reported that only self‐reports from the children were used. Half of the studies employed a combination of self‐reports and proxy reports^16^, ^18^, ^31^, ^34^, ^39^ while the other 50% studies only used proxy reports.^4^, ^35^, ^36^, ^37^, ^38^ The reason for not using only self‐reports may be because health care professionals may want to confirm the self‐reports from the communicative vulnerable children with the proxy reports from parents or their own observations of the children's nonverbal responses. This finding is supported by the recommendations for clinicians to follow during pain assessment of communicative vulnerable patients where Herr et al.^47^ proposed that clinicians should first become aware of potential causes of pain in the communicative vulnerable patient by means of proxy report or observations. As a second step, self‐report should be obtained from all patients.^47^ Regrettably, no alternative means of communication (such as augmentative and alternative communication) to self‐report were reported in any of the included studies.

From the findings of the review, both verbal and nonverbal modes of pain communication were identified in children with ASD. These findings suggest that this population do experience pain but may communicate their pain responses differently compared to children without disabilities.^24^, ^34^ This aligns with findings from Allely et al.^31^ who observed that children with ASD communicate pain differently compared to their peers without disabilities, which may cause an under‐ or overestimation of the child's pain threshold or experience. Children with ASD often present with atypical facial expressions, physiological responses, sensory processing difficulties and socioemotional responses which may be misinterpreted by observers including health care practitioners or caregivers due to the subjective nature of the observation.^48^ Additionally, their communicative vulnerability may exacerbate neuro divergent responses to pain such as increased aggressive‐ and repetitive behavior. As such, the findings suggest that apart from self‐reports from children with ASD (e.g., by using WBFPS, VAS, or a numeric rating scale) included in pain assessment,^18^, ^31^, ^39^ observations of nonverbal responses^38^, ^39^ as well as proxy reports by familiar observers such as parents and caregivers should also be considered to ensure a holistic pain assessment.^49^

Findings from this review, support that caregivers' proxy report on their children's pain cues and behaviors could also be considered as they are more familiar with the children.^5^ However, Palese et al.^38^ found that different observers, such as educators and parents scored the pain episodes of children with ASD differently because of potential biases and the context wherein they engage with the children. It might be that some observers do not understand the behavioral expressions (including verbal and nonverbal responses) of the children's responses to pain, which may influence their interpretation of the children's pain.^31^ Furthermore, observers are not always sufficiently trained about ASD^6^ and may not know how to interpret signs of pain within children with ASD due to these atypical pain responses and their communicative vulnerability.^18^ As such, consideration should be made for the reliability of proxy reports as caregivers typically perceive their child with ASD as experiencing less pain than their children without disability confirming findings from previous studies.^18^ Therefore, nonverbal responses to pain should also be considered when assessing pain within children with ASD^4^, ^35^ as established in the findings of this review.

The current review confirmed previous findings^20^, ^21^, ^50^ that children with ASD do experience pain, but their pain experience may be reflected through physiological responses (i.e., headaches, increased heartrate), difficulty in regulating sensory input (i.e., hypo or hypersensitivity),^16^, ^31^, ^34^ nonverbal communication attempts (i.e., increased facial expression, gestures)^18^, ^35^ as well as social–emotional responses (i.e., aggressive behavior, increased emotional and repetitive behavior responses)^4^, ^31^, ^35^ (see Table 2). It is often reported that children with ASD respond differently to sensory stimuli compared to children without disabilities.^16^, ^20^, ^51^ These findings may partly be due to the challenges that children with ASD experience with sensory integration and disparities in sensory experiences as described in the related diagnostic features of this disorder.^3^ However, findings of this review emphasize the importance of considering verbal and nonverbal responses during pain assessment in children with ASD^49^ through the inclusion of children's self‐reports, observational assessment tools, and proxy reports by caregivers. Holistic pain assessment can include assessing the social, psychological, spiritual, cultural, and physical pain by including all stakeholder perspectives (child, health care practitioner and caregiver).^49^, ^52^ To prevent potential biased pain assessments and the mismanagement of pain, verbal, and nonverbal responses of children with ASD and proxy reports of related stakeholders (i.e., their observers such as parents, carers, and health care practitioners), may also decrease extreme behavioral responses to pain and contribute to better pain experiences.^53^

Pediatric patients with and without disabilities often experience anxiety in health care settings.^54^ Children with ASD—similar to children without disabilities—experience anxiety during medical procedures (i.e., venepuncture,^20^, ^50^ or due to previous negative pain experiences).^48^ It is thus proposed that health care professionals provide children with ASD with additional support before or during these procedures to reduce their anxiety or distress related to these pain experiences. This support can include the provision of pictorial support (such as a schedule of the sequence of the procedure) to prepare the children with ASD and their caregivers on what to expect during the procedure.^54^, ^55^ Distraction or alternate focus during the administration of the medical procedures can also be implemented.^18^ Children who are prepared for health care experiences in a manner that is developmentally appropriate, show more positive outcomes in their behavior, recovery, and their ability to cope with stressful events.^56^, ^57^ Preparation for medical procedures furthermore leads to less emotional distress, better overall coping, clearer understanding of medical interventions and a more positive physical recovery.^58^ Kleiber and Harper^59^ conducted a meta‐analysis on 26 studies dealing with the effects of distraction or alternate focus on children's pain and distress during medical procedures. They concluded that distraction or alternate focus has a positive effect on both the children's levels of distress as well as their perception of pain.^59^ Cohen^60^ elucidates that distraction has been shown to minimize children's fear, anxiety, and pain associated with acute painful medical procedures.

### 4.1 Strengths and limitations

The findings of the current study supported the findings from the two review studies^16^, ^31^ included in this study. However, the findings of the two previous reviews were not re‐analyzed and as such the interpretation of the authors of the previous reviews might have been different than what was reported in the original studies. By definition, reporting bias may be present. Other factors that might influence children with ASD's communication of pain (e.g., their cognitive development and severity of ASD) were not always reported in selected studies included in the current review and did not receive further attention. As such, the review provides current available description of children's pain expression but should be interpreted with caution. It is further important to bear in mind the subjective responses to pain and various factors may impact on how children with ASD may communicate their pain.

### 4.2 Future research

Future research, specifically in the Global South, is needed to understand how the inclusion of different stakeholders in the pain assessment of children with ASD's pain can contribute to holistic pain assessment. The impact of psychosocial support of children with ASD as well as their caregivers on the anxiety of these children before surgery and medical interventions could also be explored.

## 5 CONCLUSION

This study supports the notion that children with ASD do experience pain, but often express their pain in neurodiverse ways when compared to children without disabilities. Based on the findings from this review, two themes were identified, namely that children with ASD communicate their pain by means of verbal and nonverbal responses to pain. Findings from the current review contradict the belief that children with ASD have a reduced pain sensitivity and do not experience pain as acutely as their peers without disabilities and acknowledges the challenges that this population encounter during a painful experience. This review may provide valuable practice implications as it reiterates the importance of the inclusion of different stakeholders in the assessment of pain to ensure appropriate pain management. Future research can focus on the contribution from the stakeholders on the pain assessment of children with ASD to alleviate intensive behavioral responses to pain and contribute to better pain experiences. The impact of psychosocial support for children with ASD to address potential anxiety before and during the administration of medical procedures is also suggested.

## CONFLICT OF INTEREST STATEMENT

The authors report no conflict of interest.

## References

[1] Bornman J . Believe that All Can Achieve. Increasing Classroom Participation in Learners with Special Support Needs. 3rd ed. Van Shaik; 2021.

[2] Restrepo BA , K , Taylor SL , et al. Developmental–behavioral profiles in children with autism spectrum disorder and co‐occurring gastrointestinal symptoms. Autism. Research. 2020;13:1778‐1789. 10.1002/aur.2354 PMC7689713 32767543

[3] American Psychiatric Association , ed. Diagnostic and Statistical Manual of Mental Disorders. 5th ed. American Psychiatric Association; 2013.

[4] Rattaz C , Dubois A , Michelon C , Viellard M , Poinso F , Baghdadli A . How do children with autism spectrum disorders express pain? A comparison with developmentally delayed and typically developing children. Pain. 2013;154(10):2007‐2013. 24040973 10.1016/j.pain.2013.06.011

[5] Scarpinato N , Bradley J , Kurbjun K , Bateman X , Holtzer B , Ely E . Caring for the child with an autism Spectrum disorder in the acute care setting. J Spec Pediatr Nurs. 2010;15(3):244‐254. 20618639 10.1111/j.1744-6155.2010.00244.x

[6] Arnold B , Elliott A , Laohamroonvorapongse D , Hanna J , Norvel D , Koh J . Autistic children and anesthesia: is their perioperative experience different? Pediatric Anesthesia. 2015;25:1103‐1110. 26338278 10.1111/pan.12739

[7] Jolly AA . Handle with care: top ten tips a nurse should know before caring for a hospitalized child with autism Spectrum disorder. Pediatr Nurs. 2015;41(1):11‐16, 22. 26281270

[8] Costello J , Patak L , Pritchard J . Communication vulnerable patients in the pediatric ICU: enhancing care through augmentaitve and alternative communication. J Pediatr Rehabil Med. 2010;3:289‐301. 21791863 10.3233/PRM-2010-0140

[9] Costello J , Santiago R , Blackstone SW . Pediatric acute and intensive care in hospitals. In: Blackstone SW , Beukelman DR , Yorkson KM , eds. Patient Provider Communication: Roles for Speech‐Language Pathologists and Other Health Care Professionals. Plural Publishing; 2015:187‐215.

[10] Raja SN , Carr DB , Cohen M , et al. The revised International Association for the Study of Pain definition of pain: concepts, challenges, and compromises. Pain. 2020;161:1976‐1982. 32694387 10.1097/j.pain.0000000000001939 PMC7680716

[11] Schudlo LC , Anagnostou E , Chau T , Doyle‐Thomas K . Investigating sensory response to physical discomfort in children with autism spectrum disorder using near‐infrared spectroscopy. PloS One. 2021;16(9):e0257029. 34478466 10.1371/journal.pone.0257029 PMC8415580

[12] Pediatrics AAo . Policy statement—child life services. Pediatrics. 2014;133(5):1471‐1478. 10.1542/peds.2014-0556 24777212

[13] Björkman B , Golsäter M , Enskär K . Children's anxiety, pain, and distress related to the perception of care while undergoing an acute radiographic examination. J Radiol Nurs. 2014;33(2):69‐78.

[14] Herr K , Coyne PJ , McCaffery M , Manworren R , Merkel SI . Pain assessment in the patient unable to self‐report: position statement with clinical practice recommendations. Pain Manag Nurs. 2011;12(4):230‐250. 22117755 10.1016/j.pmn.2011.10.002

[15] Twycross A , Voepel‐Lewis T , Vincent C , Franck LS , von Baeyer CL . A debate on the proposition that self‐report is the gold standard in assessment of pediatric pain intensity. Clin J Pain. 2015;31(8):707‐712. 25370143 10.1097/AJP.0000000000000165

[16] Moore DJ . Acute pain experience in individuals with autism spectrum disorders: a review. Autism. 2015;19(4):387‐399. 24687688 10.1177/1362361314527839

[17] Oberlander TF , Symons FJ . Pain in Children and Adults with Developmental Disabilities. Paul H Brookes Pub Co; 2006.

[18] Ely E , Chen‐Lim ML , Carpenter KM , Wallhauser E , Friedlaender E . Pain assessment of children with autism spectrum disorders. J Dev Behav Pediatr. 2016;37(1):53‐61. 26703326 10.1097/DBP.0000000000000240

[19] Garrick A , Lee ML , Scarffe C , et al. An Australian cross‐sectional survey of parents' experiences of emergency department visits among children with autism spectrum disorder. J Autism Dev Disord. 2021;1‐15:2046‐2060. 10.1007/s10803-021-05091-9 34061310

[20] Nadar R , Oberlander TF , Chambers CT , Craig KD . The expression of pain in children with autism. Clin J Pain. 2004;20:88‐97. 14770048 10.1097/00002508-200403000-00005

[21] Tordjman S , Anderson GM , Botbol M , et al. Pain reactivity and plasma β‐endorphin in children and adolescents with autistic disorder. PLoS One. 2009;4(8):e5289. 19707566 10.1371/journal.pone.0005289 PMC2728512

[22] Bornman J , Tönsing KM . In: Landsberg E , Kruger D , Swart E , eds. Augmentative and Alternative Communication. 4th ed. Van Schaik; 2019.

[23] Corcoran N . Theories and Models in Communicating Health Messages. SAGE Publications; 2007:5‐31.

[24] Knoll A , McMurtry CM , Chambers CT . Pain in children with autism spectrum disorder: experience, expression, and assessment. Pediatric Pain Letter. 2013;15(2):23‐28.

[25] Craig KD , Lilley CM , Gilbert CA . Social barriers to optimal pain management in infants and children. Clin J Pain. 1996;12:232‐242. 8866164 10.1097/00002508-199609000-00011

[26] Dubois A , Rattaz C , Pry R , Baghdadli A . Autism and pain‐a literature review. Pain Res Manag. 2010;15(4):245‐253. 20808970 10.1155/2010/749275 PMC2935725

[27] Munn Z , Peters MDJ , Stern C , Tufanaru C , McArthur A , Aromataris E . Systematic review or scoping review? Guidance for authors when choosing between a systematic or scoping review approach. BMC Med Res Methodol. 2018;18:1‐7. 30453902 10.1186/s12874-018-0611-x PMC6245623

[28] Tricco AC , Lille E , Zarin W , et al. PRISMA extension for scoping reviews (PRISMA‐ScR): checklist and explanation. Ann Intern Med. 2018;169:467‐473. 30178033 10.7326/M18-0850

[29] Peters MDJ , Marnie C , Colquhoun H , et al. Scoping reviews: reinforcing and advancing the methodology and application. BMC. 2021;10:263. 10.1186/s13643-021-01821-3 PMC8499488 34625095

[30] Peters MDJ , Godfrey CM , McInerney P , Soares C , Khalil H , Parker D . The Joanna Briggs Institute reviewers' Manual 2015: Methodology for JBI Scoping Reviews. The Joanna Briggs Institute; 2015.

[31] Allely CS . Pain sensitivity and observer perception of pain in individuals with autistic spectrum disorder. Scientific World J. 2013;1‐20:1‐20. 10.1155/2013/916178 PMC3697411 23843740

[32] Friese S . Qualitative Data Analysis with ATLAS. Sage; 2019.

[33] Braun V , Clarke V . One size fits all? What counts as quality practice in (reflexive) thematic analysis? Qual Res Psychol. 2021;18(3):328‐352.

[34] Clarke C . Autism spectrum disorder and amplified pain. Case reports. Psychiatry. 2015;2015:1‐4. 10.1155/2015/930874 PMC4438170 26064754

[35] Prosperi M , Santocchi E , Muratori F , et al. Vocal and motor behaviors as a possible expression of gastrointestinal problems in preschoolers with autism spectrum disorder. BMC Pediatr. 2019;19(1):1‐10. 31779607 10.1186/s12887-019-1841-8 PMC6883656

[36] English MS , Tenenbaum EJ , Levine TP , Lester BM , Sheinkopf SJ . Perception of cry characteristics in 1‐month‐old infants later diagnosed with autism spectrum disorder. J Autism Dev Disord. 2019;49(3):834‐844. 30361941 10.1007/s10803-018-3788-2 PMC10266897

[37] Dubois A , Michelon C , Rattaz C , Zabalia M , Baghdadli A . Daily living pain assessment in children with autism: exploratory study. Res Dev Disabil. 2017;62:238‐246. 28089432 10.1016/j.ridd.2017.01.003

[38] Palese A , Conforto L , Meloni F , et al. Assessing pain in children with autism spectrum disorders: findings from a preliminary validation study. Scand J Caring Sci. 2021;35(2):457‐467. 32311779 10.1111/scs.12857

[39] Fitzpatrick R , McGuire BE , Lydon HK . Improving pain‐related communication in children with autism spectrum disorder and intellectual disability. Paediatric and neonatal. Pain. 2022;4:22‐32. 10.1002/pne2.12076 PMC8975218 35546916

[40] Braun V , Clarke V . Successful Qualitative Research: A Practical Guide for Beginners. SAGE; 2013.

[41] Review WP . Global South Countries 2023. 2023.

[42] Hamadeh N , Van Rompaey C , Metreau E , Eapen SG . New World Bank country classifications by income level 2022–2023. 2022.

[43] Hashemi G , Kuper H , Wickenden M . SDGs, inclusive health and the path to universal health coverage. Disabil Global South. 2017;4(1):1088‐1111.

[44] Masuku K , Bornman J , Johnson E . Access to healthcare for persons with disabilities in Eswatini: a triadic exploration. African Disabil Rights Yearbook. 2021;9:138‐159.

[45] Hashemi G , Wickenden M , Bright T , Kuper H . Barriers to accessing primary healthcare services for people with disabilities in low and middle‐income countries, a meta‐synthesis of qualitative studies. Disabil Rehabil. 2022;44(8):1207‐1220. 32956610 10.1080/09638288.2020.1817984

[46] Masuku K , Bornman J , Johnson E . Analyzing Eswatini's national disability policy reforms: access to health care implications for citizens with disabilities. J Disabil Policy Stud. 2023;10442073231165775.

[47] Herr K , Coyne PJ , Ely E , Gélinas C , Manworren RCB . Pain assessment in the patient unable to self‐report: clinical practice recommendations in support of the ASPMN 2019 position statement. Pain Manag Nurs. 2019;20(5):404‐417. 31610992 10.1016/j.pmn.2019.07.005

[48] Bottos S , Chambers CT . The epidemiology of pain in developmental disabilities. In: Orlander TM , Symons FJ , eds. Pain in Children and Adults with Developmental Disabilities. Paul Brookes Publishing Company; 2006:67‐87.

[49] Brant JM . Holistic total pain management in palliative care: cultural and global considerations. Palliative Med Hosp Care Open J. 2017;1:S32‐S38.

[50] Messmer RL , Nader R , Craig KD . Brief report: judging pain intensity in children with autism undergoing venepuncture: the influence of facial activity. J Autism Dev Disord. 2008;38(7):1391‐1394. 18161016 10.1007/s10803-007-0511-0

[51] Klintwall L , Holm A , Eriksson M , Carlsson LH , Olsson MB , Hedvall A . Sensory abnormalities in autism: a brief report. Res DevStud. 2011;32(2):795‐800. 10.1016/j.ridd.2010.10.021 21111574

[52] Phenwan T . Relieving total pain in an adolescent: a case report. BMC Res Notes. 2018;11(1):265. 29720253 10.1186/s13104-018-3368-8 PMC5930844

[53] Rattaz C , Dubois A , Baghdadli A . How do people with autism spectrum disorders (ASD) experience pain? In: Battaglia AA , ed. An Introduction to Pain and its Relation to Nervous System Disorders. Wiley Online Library; 2016:295‐315.

[54] Chebuhar A , McCarthy AM , Bosch J , Baker S . Using picture schedules in medical settings for patients with an autism spectrum disorder. J Pediatr Nurs. 2013;28(2):125‐134. 22742928 10.1016/j.pedn.2012.05.004

[55] Vantaa Benjaminsson M , Thunberg G , Nilsson S . Using picture and text schedules to inform children: effects on distress and pain during needle‐related procedures in nitrous oxide sedation. Pain Res Treat. 2015;2015:478503. 26798514 10.1155/2015/478503 PMC4700196

[56] Gaynard L , Wolfer J , Goldberg J , Thompson R , Redburn L , Laidley L . Psychosocial Care of Children in Hospital: A Clinical Practice Manual from the ACCH. Child Life Council; 1998.

[57] Thompson RH . The Handbook of Child Life: A Guide to Pediatirc Psychosocial Care. Thomas Books; 2009.

[58] Romito B , Jewell J , Jackson M ; AAP COMMITTEE ON HOSPITAL CARE ; ASSOCIATION OF CHILD LIFE PROFESSIONALS . Child Life Services. Pediatrics. 2021;147(1):e2020040261. 33372119 10.1542/peds.2020-040261

[59] Kleiber C , Harper DC . Effects of distraction on children's pain and distress during medical procedures: a meta‐analysis. Nurs Res. 1999;48(1):44‐49. 10029401 10.1097/00006199-199901000-00007

[60] Cohen LL . Behavioral approaches to anxiety and pain management for pediatric venous access. Pediatrics. 2008;122(Supplement_3):S134‐S139. 18978007 10.1542/peds.2008-1055f

